# An efficient and cost-effective method for primer-induced nucleotide labeling for massive sequencing on next-generation sequencing platforms

**DOI:** 10.1038/s41598-019-38996-8

**Published:** 2019-02-28

**Authors:** Junjie Guo, Tao Cheng, Han Xu, Yide Li, Jie Zeng

**Affiliations:** 1 Research Institute of Tropical Forestry, Chinese Academy of Forestry, Longdong, Guangzhou 510520 China; 2 Berry Genomics Corporation Limited, Changping, Beijing 102200 China; 3 Jianfengling National Key Field Research Station For Tropical Forest Ecosystem, Hainan, 572500 China

## Abstract

Next generation sequencing (NGS) technologies play a powerful role in the preparation of large DNA databases such as DNA barcoding since it can produce a large number of sequence reads. Here we demonstrate a primer-induced sample labeling method aiming at sequencing a large number of samples simultaneously on NGS platforms. The strategy is to label samples with a unique oligo attached to the 5′-ends of primers. As a case study, 894 unique pentanucleotide oligoes were attached to the 5′-ends of three pairs of primers (for amplifying ITS, *mat*K and *rbc*L) to label 894 samples. All PCR products of three barcodes of 894 samples were mixed together and sequenced on a high throughput sequencing platform. The results showed that 87.02%, 89.15% and 95.53% of the samples were successfully sequenced for *rbc*L, *mat*K and ITS, respectively. The mean ratio of label mismatches for the three barcodes was 5.68%, and a sequencing depth of 30 ×to 40× was enough to obtain reliable sequences. It is flexible to label any number of samples simply by adjusting the length of oligoes. This easy, reliable and cost efficient method is useful in sequencing a large number of samples for construction of reference libraries for DNA barcoding, population biology and community phylogenetics.

## Introduction

Since the DNA barcoding concept was proposed by Hebert *et al*.^1^ DNA-based taxonomic identification has become a very important tool, and great progress has been made on the improvement. The mitochondrial gene COI^2^ was developed as standard barcode of animals. Four plastid DNA regions, *rbc*L, *mat*K, *trn*H*-psb*A^3–6^ and *ycf1*^7^, and the nuclear DNA region ITS are used as the core barcodes for plants. For fungi, the ITS region is a standard^8^. A two-step barcoding strategy was suggested for protist identification, and V4 region of the 18S ribosomal DNA was the basic barcode, with one or several additional barcodes specific to different taxa^9^. No matter how well the barcodes are designed, their applications are depended on reliable reference libraries with high species coverage. Therefore, cost efficient sequencing methods are a primary concern when a large number of samples are analyzed.

In the past decade, next-generation sequencing (NGS) technologies have revolutionized DNA sequencing, and provided a new and exciting platform in evolutionary, biomedical and agronomical studies^10–14^. NGS produces millions or billions of DNA sequences in a single run on the platforms such as Roche 454^15^, Illumina^16^ and ABI SOLiD^17^, and makes DNA sequencing more cost effective and fast^18^. Because of the advantages of this technology, the platforms acquired great attentions in whole genome *de novo* sequencing^16,19–21^, whole genome resequencing^22^, transcriptome sequencing^13,23^, and small RNAs sequencing^24^, etc. However, the studies involving a large number of samples have so far benefited very little from such technical developments due to the limited ability in sample identifications. Sample identification is a big problem to be solved in adoption of NGS. Recently, some attempts have been made to sequence a number of samples at once using DNA-tagged parallel sequencing methods, with unique labels or tags linked to each sample in order to distinguish them^25–29^. Although these attempts made accurate identification of both gene fragments and samples, and made NGS more scalable and efficient, there were still some drawbacks. One such a drawback is that only a small number of samples (less than 300) can be identified at once using two-nucleotide tags in some cases, and thus cannot meet the demand for massive sequencing. Another is that long tags (8–10 bp) in turn result in heavy costs when thousands of primers need to be synthesized. There is conflict between labeling a large number of samples and costing efficiently according to the principle that differences of at least four nucleotides should exist between paired labels.

Here, we present an easier and more cost-effective method to label a large number of samples for NGS. We describe how the plant DNA barcodes of ITS, *rbc*L and *mat*K of 894 pant samples were sequenced simultaneously on the Roche 454 plus platform. This method is applicable on any other NGS platforms.

## Results

### DNA labels and their effects on PCR

A total of 960 unique labels were designed, and 894 designed labels were used to label 894 samples in this study. To test the possible effects of the addition of labels on PCR success, both labeled and unlabeled primers were used to simultaneously amplify all three fragments, ITS, *mat*K, and *rbc*L, in 96 randomly selected samples. There were 88.19%, 88.54% and 97.22% successfully amplified samples with labeled primers for ITS, *mat*K and *rbc*L, respectively. With unlabeled primers, 86.81%, 87.50% and 97.22% for ITS, *mat*K and *rbc*L were successfully amplified, respectively. No significant differences (*p* ≥ 0.05) in PCR success percentage were observed between both types of primers for each barcode, while significant differences exist among the three barcodes (*p* < 0.05).

### Raw data information from GS FLX plus platform

In total, 894 samples were sequenced for the three barcodes on the GS FLX plus platform in a single run. A total of 791,690 reads were generated (Table 1). ITS, *mat*K and *rbc*L had 183,379, 268,877 and 339,434 reads, respectively. The average length of all reads including labels was 590 bp, and the average read lengths of ITS, *mat*K and *rbc*L were 570 bp, 610 bp and 586 bp, respectively. The average lengths of forward and reverse reads were approximately equal for each region.

**Table 1:** The raw data and labels performance information from Roche 454 GS FLX plus platform sequencing.

Barcode	Total no. of samples	Assigned no. of samples	Reads information					sequencing depth*
			Total no.	Assigned no. (%)	Forward assigned no. (%)	Reverse assigned no. (%)	Not assigned no. (%)	
ITS	894	890	183,379	159,545 (87.00%)	78,348 (42.72%)	81,197 (44.28%)	23,834 (13.00%)	205
*mat*K	894	884	268,877	229,620 (85.40%)	113,986 (42.39%)	115,634 (43.01%)	39,257 (14.60%)	300
*rbc*L	894	873	339,434	298,163 (87.84%)	129,881 (38.26%)	168,282 (49.58%)	41,271 (12.16%)	379
Total	2,682	2,647	791,690	687,328 (86.81%)	322,215 (40.70%)	365,113 (46.11%)	104362 (13.19%)	295

*Sequencing depth = total reads / total samples.

### Sequencing depth and reliability

In a single run for 894 samples, the sequencing depths ranged from 205× for ITS to 379× for *rbc*L with a mean of 295× (Table 1). To test the reliability of sequences, 141 samples were duplicated and sequenced. Among all 141 comparisons, 99 (70.2%) had identical sequences, 38 (27.0%) had one to five gaps, and 4 (2.8%) had one mismatch. We further conducted Sanger sequencing of the three barcodes for 92 out of the 141 samples, and found out that the consensus sequences from 454 sequencing were the same as those from Sanger sequencing.

### Assigned ability of pentanucleotide labels

In total, 687,328 out of 792,690 reads (86.81%) were assignable to 894 samples (Table 1). The percentages of assigned reads varied from 85.40% to 87.84% among the three barcodes. The number of assigned forward vs reverse reads were 78,348 (42.72%) vs 81,197 (44.28%), 113,986 (42.39%) vs 115,634 (43.01%) and 129,881 (38.26%) vs 168,282 (49.81%) in ITS, *mat*K and *rbc*L, respectively. Chi squared (χ^2^) test strongly rejected equal distributions among the different labels for all three barcodes (ITS: χ^2^ = 602.58, *p* < 0.01; *mat*K: χ^2^ = 946.05, *p* < 0.01; *rbc*L: χ^2^ = 870.77, *p* < 0.01).

### Ratio of sequencing success with pentanucleotide labels

Sequencing successes varied among *rbc*L, *mat*K and ITS. Of 894 samples, 778 (87.02%), 797 (89.15%) and 854 (95.53%) samples were successfully sequenced for *rbc*L, *mat*K and ITS, respectively. Sequencing failure is a common problem due to PCR failure. For the successfully sequenced samples, there were specific amplifications of 96.13% for *rbc*L, 93.95% for *mat*K and 78.75% for ITS. Of the error reads, 2.19%, 1.82% and 6.02% of reads resulted from microorganism contaminations (P_S_), and 1.68%, 4.23% and 15.23% of reads from label mismatch (P_M_) in *rbc*L, *mat*K and ITS, respectively. The weighted mean ratio of label mismatches for the three barcodes was 5.68%.

### Relationship between sequence accuracy and sequencing depth

**Figure 1:**
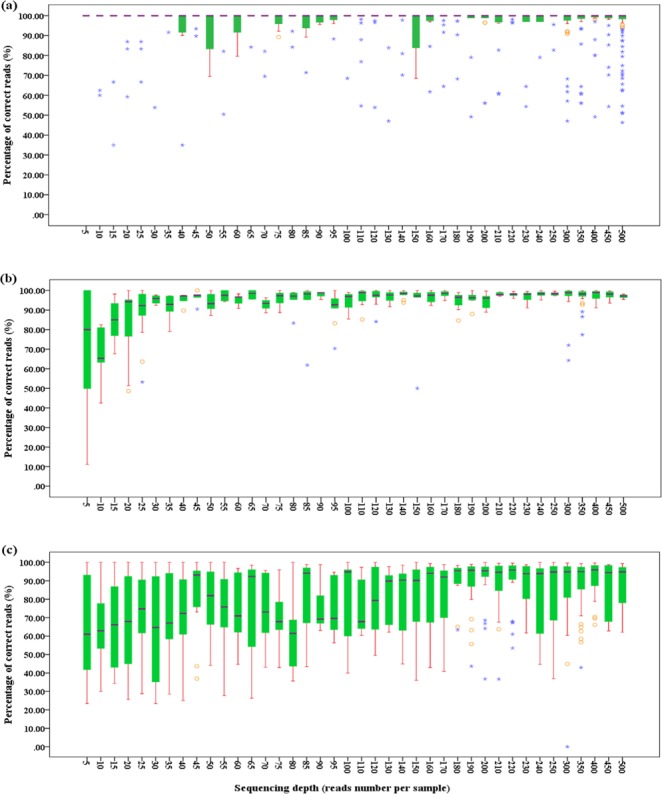
The percentage of correct reads at different sequencing depths. (**a**–**c)** Stand for *rbc*L, *mat*K and ITS, respectively.

The ratio of correct reads for each sample increased with the increase of sequencing depth of both strands for all the three regions, *rbc*L, *mat*K and ITS (Fig. 1), while among these regions, there were obvious differences in the ratios of correct reads. No consensus sequence difference was observed from 5× to 500× for *rbc*L. For *mat*K, remarkable increases of sequence correctness were observed for sequencing depths of less than 30×, while little changes appeared above 30×. Therefore, 30× is the most cost efficient sequencing depth for *mat*K. A sequencing depths of 85× is required for ITS because ITS is the most variable barcode suffering from adverse factors such as multiple copies and fungus contaminations, etc.

**Figure 2:**
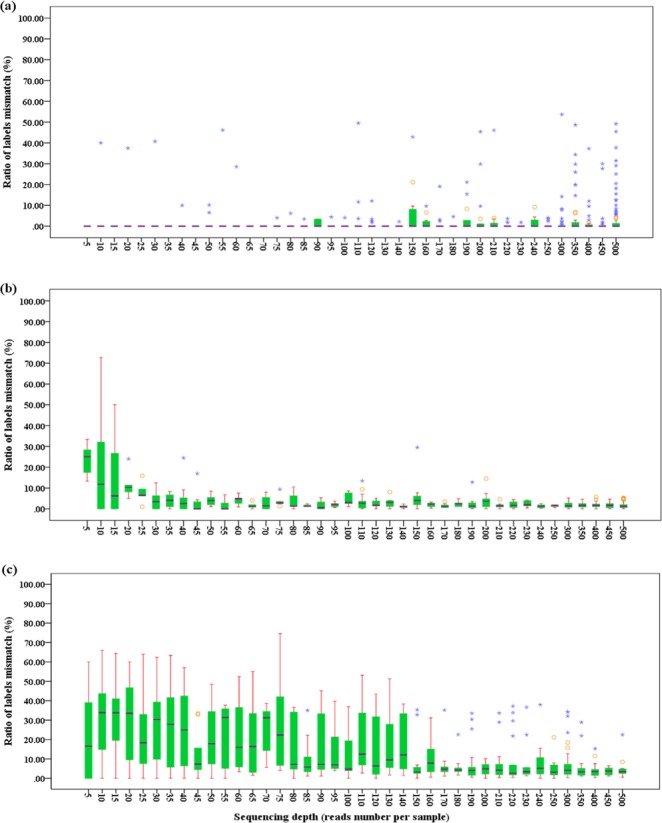
The ratio of labels mismatched at different sequencing depths. (**a**–**c)** Stand for *rbc*L, *mat*K and ITS, respectively.

We have also calculated the percentage of error reads caused by label mismatch for each sample and assessed the effect of sequencing depths. We found that the percentage of error reads for *mat*K and *ITS* decreased with the increase of sequencing depths (Fig. 1). The sequencing depths of 30× and 80× were the inflection points for the percentage of error reads caused by label mismatch for *mat*K and ITS regions, respectively. For *rbc*L gene, while the percentage of error reads changed irregularly, it was very low with the mean of 1.68% from sequenced depths 5× to 500× (Fig. 2).

## Discussion

The primers linked with pentanucleotide labels showed a good performance in the PCR and pyrosequencing process in the present study. Of all the obtained reads, 86.81% reads were labeled and assignable to all 894 samples, and 87.02%, 89.15% and 95.53% samples were successfully sequenced for *rbc*L, *mat*K and ITS, respectively. The sequencing accuracy was similar to other studies including 84%, 88% and 93.7% for Galan *et al*.^27^, Bybee *et al*.^28^ and Shokralla *et al*.^29^, respectively. Although our pentanucleotide labels had no negative effect on the PCR success, the χ^2^ test showed that the number of reads were significantly different among barcodes. This was similar to the study of Binladen *et al*.^25^. This phenomenon could be explained by the unequal amount of PCR products sequenced. In the present study, the PCR products of 894 samples were not produced at equimolar concentrations. Even though the three barcodes were sequenced with an equimolar concentration, it might produce unequal reads due to uncontrollable random factors. In Binladen *et al*.’s^25^ study with 13 samples and one gene, sequencing PCR products of an equimolar concentration also produced unequal number of reads for each sample. Therefore, differences in amplicon concentrations might not be the main cause for bias of the read numbers.

In our strategy, pentanucleotide labels with at least one bp difference were used to identify reads and assign them into each sample accordingly. It is quite different from those of the previous studies. In Bybee *et al*.’s^28^ study, the labels were designed strictly based on differences of decanucleotide at least 4 nucleotides from each other in order to repair two or fewer ambiguities. Shokralla *et al*.^29^ reported that the labels should differ in at least 2 nucleotides from each other, and the purposes for this were to make the ratio of primer mismatch of sequencing reads as low as possible and to keep high accuracy in assigning samples. The ratio of label mismatches was thus the primary concern for designing 5′– end labels. In our study, the different levels of primer mismatches existed in three barcodes. The smallest ratio of label mismatches was in *rbc*L, and the biggest one was in ITS. This might be explained due to the biases in calculating the ratio of label mismatches. Because *rbc*L sequences had the slowest evolution rate among the three regions, it had the lowest divergence at the species level. Consequently, it might have a lower calculated ratio of label mismatches. In contrast, ITS had a calculated higher ratio of label mismatches probably due to sequence duplication and microorganism contamination during sequencing. The highly specific ITS primers for plant would be developed so as to improve the sequencing accuracy in the future. The *mat*K sequences originate from the plant chloroplast DNA, and thus its calculated concentration was less affected by microorganism contamination. The mean ratio of primer mismatches was 5.68% for these three regions, which was similar to those for the labels with more than one bp differences, such as 6.8% reported in Binladen *et al*.^25^ and 7.8% in O’NEILL *et al*.^30^.

More nucleotide differences would make the labels longer and result in higher costs of label synthesis. We designed pentanucleotides with at least one bp difference to label 960 samples which were much more than those in the previous studies^25,27–30^. Of course, 4^6^ = 4096 samples could be labeled theoretically if hexnucleotides were used, while it is not a better way due to high cost of their label synthesis in practice. In our case, the cost of a single run on Roche 454 GS FLX plus platform was $13,700 (price in 2015 as reference, with the cost of label synthesis not included). The mean cost per locus per sample is $3.83, a cost similar to Sanger sequencing in China (the cheapest price is $4 per locus per sample). However, when one million usable reads and a depth of 30× are concerned, 8,333 samples could be sequenced in a single run, and the cost per sample per locus would be only $0.41 (costs for library constructions not considered). Taking 4 × 960 samples as an example without extra charges, the cost per sample per locus would be $0.89. To sequence the same three barcodes of the same number of samples using Sanger sequencing method, the cheapest cost would be $61,440. This labeling strategy can be further upgraded by introducing other labeling methods, which would significantly reduce the costs on primers and makes this strategy more cost-efficient.

There might be some practical concerns toward the application of NGS on multiple sample sequencing. The first one is the depth of sequencing. Our empirical data backed up with *mat*K showed that 30× to 40× is enough to obtain reliable sequences. The second concern is the accuracy of sequences. The biggest problem is slippage of poly-structure which is a flaw of almost all NGS platforms. This kind of problem does not bring troubles to DNA barcoding or phylogenetic reconstruction because in these applications the gaps are usually treated as missing. The third concern is the efficiency. The sequencing efficiency of NGS is in general much higher than conventional Sanger sequencing method. It usually takes a few days for bioinformatic treatment of reads using TNAssembler pipeline programs, while sequence edition takes a lot of time to check sequences from conventional Sanger sequencing. If multiple PCRs were used, the experimental procedures would be even speeded up significantly.

Although Roche 454 GS FLX plus was used for sequencing in our study, our DNA labeling method was developed aiming at all high throughput platforms. The determinant factor for application of any sequencing platform is only the fragment length which is dependent on primer pairs. If internal primers are used to amplify the fragments shorter than 500 bp, Illumina MiSeq even HiSeq platform can be used and much more samples could be sequenced simultaneously (http://systems.illumina.com/systems/miseq.ilmn).

In conclusion, our study provides a method of labeling samples by adding five nucleotides to the 5′-end of PCR primers. This strategy is applicable to any number of samples and genes for any NGS platforms. For practical reasons, a set of 384 to 960 unique labels linked to universal primers works better together with other labels used for library constructions. This approach has been proven quick and cost-efficient, and will contribute greatly to not only DNA barcoding, but also community phylogenetics and population genetics.

## Methods

### DNA label design and primer labeling

**Figure 3:**
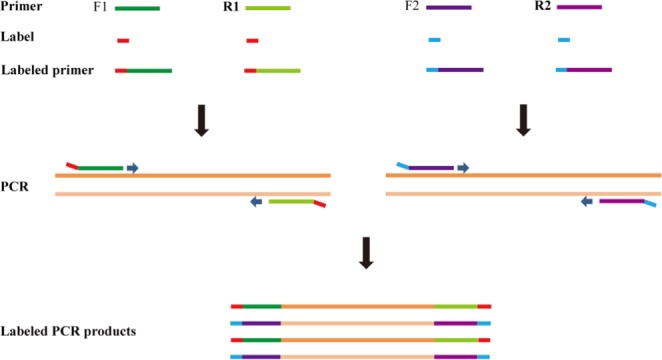
Schematic diagram of labeling by 5′-end labeled primers. F1 and F2 refer to forward primers, and R1 and R2 to reverse primers for different markers.

The principles to design DNA labels are: (i) to label a medium number of samples with lower costs in primer synthesis; (ii) no or little side effects on PCR; (iii) sequences of both forward and reverse strands amplified by primer are usable; and (iv) at least one bp difference from each other. We designed pentanucleotide labels and tagged them to primers (Fig. 3). The five nucleotides could produce 1024 (4^5^) labels theoretically. To reduce possible side effects, all polynucleotides (such as AAAAA, CCCCC, etc.) and most of hairpins (such as AAATT, CCAGG, etc.) were not used. We eventually selected 960 unique labels at the 5′ends of both forward and reverse primers and used 894 of them to label 894 samples. Three pairs of primers with DNA barcodes for ITS^31^, *mat*K^32^ and *rbc*Lb^33^ were labeled, synthesized and used in this study (Table S1).

### Plant materials

A total of 894 samples belonging to 293 species, 164 genera, 76 families were collected from a tropical forest dynamics plot at Jianfengling National Key Field Research Station on Hainan Island, China (Table S2). Among them 141 individuals were sampled twice to test the reliability of the sequencing method. Leaves of all samples were dried quickly in silica gel with a weight ratio of leaf / silica = 1/10.

### DNA extraction and PCR amplication

Total DNA was extracted from dried leaves following Zeng *et al*.^34^. PCRs were conducted in a 30 μL reaction volume containing 2 μL DNA template (about 50 ng/μL), 10.2 μL H_2_O, 1.4 μL DNA labeled forward primer (5 μM), 1.4 μL DNA labeled reverse primer (5 μM) and 15.0 μL 2× Taq Plus PCR Master Mix (Tiangen Biotech (Beijing) Co., Ltd, China). PCR amplification was performed in a Master cycler Gradient Thermal Cycler (Eppendorf) with the following program: initial denaturation at 94 °C for 4 minutes, followed by 35 cycles of 94 °C for 30 seconds, 52 °C for 30 seconds and 72 °C for 90 seconds, and a final extension of 10 minutes at 72 °C. PCR products were checked on 1% agarose gels.

### Sequencing on 454 GS FLX plus platform

The PCR products of the same barcode for different samples were pooled, purified by running a 2% agarose gel electrophoresis. The DNA retrieved from the agarose gel was quantified using a Nanodrop ND-1000 (Nanodrop Technologies). The quantified amplicons of 3 barcodes were mixed together to construct a library for sequencing, and a single run was performed on Roche 454 GS FLX plus platform following manufacturer’s instructions.

### Sequence assignment and basic statistics

The quality of reads from Roche 454 GS FLX plus platform were checked using NGSQCtoolkit version 2.3.3^35^ with the default settings. Reads shorter than 100 bp were discarded and the sequence quality lower than Q20 were trimmed. The reads were first sorted into barcodes according to the primer sequences, and then a name or number was given to each read according to the label using the TNAssembler pipeline (http://sourceforge.net/projects/tnassembler/). The repetitive reads of each sample were assembled into contig using the Usearch^36^ function of TNAssembler, this contig was imported into Sequencer and assembled again with the 97% minimum match and 90 bp minimum overlap. The sense and anti-sense sequences were assembled separately. One or several consensus sequences were extracted based on contigs for each sample, and were named differently for distinguishing each other.

The accuracy of both the sense and anti-sense consensus sequences was estimated with phylogenetic analysis (maximum parsimony) and BLAST^37^ methods since no sequence existed in DNA database of NCBI for some species. If the consensus sequence matched one of the following two conditions, we considered this sequence to be correct. (i) Based on the consensus sequence, each sample was aligned to samples of its sibling species and other samples of the same species in phylogenetic analysis; and (ii) the consensus sequence was subjected to BLAST analysis and had hits with an identical E-score belonging to the same taxon. The correct sense and anti-sense sequences were merged into unique correct sequence of the sample. We considered all residual consensus sequences as errors. If there were n (n ≥ 1) correct consensus sequences belonging to the sequenced sample, we considered that the sample was sequenced successfully. We also conducted Sanger sequencing for 92 samples from the 141 duplicated samples so as to test whether we had obtained the true sequence of them. The ratio of samples sequenced successfully (R) was calculated by the following equation:

1 $${\rm{R}}( \% )={{\rm{N}}}_{{\rm{1}}}{/{\rm{N}}}_{{\rm{0}}}\times 100$$

Where N_1_ was the number of samples sequenced correctly, and N_0_ was the number of all samples detected.

Generally, the error reads were produced with the following three causes: E1, the label mismatch occurred when PCR and sequencing; E2, the DNA templates were cross-contaminated in the process of DNA extraction and PCR; and E3, the sampled plant materials were contaminated with microorganisms and epiphytes, e.g. fungi, insects, and lichens etc. E3 could be excluded by phylogenetic analysis (maximum parsimony) and BLAST methods. If the reads were not the sampled species, the errors were considered to belong to E3. But it was so difficult to distinguish E1 and E2 that we merged E2 into label mismatch (E1).

In order to quantify the effects of the above three causes on read errors, we analyzed the read composition of the samples sequenced successfully. We calculated the percentage of correct reads (P) by Equation (2) for each barcode:

2 $${\rm{P}}( \% )={{\rm{n}}}_{{\rm{1}}}{/{\rm{n}}}_{{\rm{0}}}\times {\rm{100}}$$

where n_1_ referred to the correct reads number and n_0_ to total reads number each barcode.

The ratio of label mismatches (P_M_) and the ratio of reads contaminated with microorganism (P_S_) were calculated according to Equations (3) and (4), respectively:

3 $${{\rm{P}}}_{{\rm{M}}}( \% )={{\rm{N}}}_{{\rm{E}}}{/{\rm{n}}}_{{\rm{0}}}\times {\rm{100}}$$

4 $${{\rm{P}}}_{{\rm{S}}}( \% )={{\rm{N}}}_{{\rm{S}}}{/{\rm{n}}}_{{\rm{0}}}\times {\rm{100}}$$

where N_E_ referred to the number of error reads coming from E2 and E3, N_S_ to the number of error reads coming from E1, and n_0_ to total number of reads for each barcode.

The mean ratio of label mismatches for the three barcodes was by Equation (5):

5 $${{\rm{P}}}_{{\rm{w}}}( \% )=({{\rm{N}}}_{{\rm{ER}}}+{{\rm{N}}}_{{\rm{EM}}}+{{\rm{N}}}_{{\rm{EI}}}){/{\rm{n}}}_{{\rm{A}}}\times {\rm{100}}$$

where N_ER_, N_EM_ and N_EI_ referred to the number of N_E_ for rbcL, matK and ITS, respectively; and n_A_ to the total number of reads for three barcodes.

In order to explore whether the read number could affect sequencing successfulness of a sample or not, the percentage of correct reads (P_C_) was calculated by Equation (6) for each sample:

6 $${{\rm{P}}}_{{\rm{C}}}( \% )={{\rm{N}}}_{{\rm{C}}}{/{\rm{n}}}_{{\rm{s}}}\times {\rm{100}}$$

where N_C_ referred to the number of correct reads and n_s_ to total number of reads for each sample.

### DNA sequences

All sequence data will be deposited in the National Center for Biotechnology Information (NCBI) Sequence Read Archive (accession no. SRR8325810, SRR8325811 and 372 SRR8325812).

## Supplementary information

Supplementary information
