# Supplementary material for: An efficient and cost-effective method for primer-induced nucleotide labeling for massive sequencing on next-generation sequencing platforms
Source: Sci Rep. 2019 Feb 28;9:3125. doi: 10.1038/s41598-019-38996-8 (PMC6395609; doi:10.1038/s41598-019-38996-8)
Supplement: Supplementary file 1 — Supplementary information [file 41598_2019_38996_MOESM1_ESM.pdf]

An efficient and cost-effective method for primer-induced nucleotide labelling for massive sequencing on next-generation sequencing platforms

Junjie Guo[1,3,†], Tao Cheng[2,†], Han Xu[1,3], Yide Li[1,3*], Jie Zeng[1,3,*]

Supplementary information

Table S1. The information of three barcodes used in this study

Name	Sequences(5'-3')	Ta(℃)
ITS	F:TCCTACCGATTGAATGRTCCGG	52
	R:AGCCTTRGAHGGRATTTACC	
*mat*K	F:CCCRTYCATCTGGAAATCTTGGTTC	52
	R:GCTRTRATAATGAGAAAGATTTCTGC	
*rbc*L	F:AGACCTWTTTGAAGAAGGTTCTGT	52
	R:TCGGTYAGAGCRGGCATATGCCA	

Table S2. The sequences of all 960 pentanucleotide labels

Name	Sequences(5'-3')
H001	AACAA
H002	AACAC
H003	AACAG
H004	AACAT
H005	AACCA
H006	AACCG
H007	AACCT
H008	AACGA
H009	AACGC

H010	AACGG
H011	AACGT
H012	AACTA
H013	AACTC
H014	AACTG
H015	AACTT
H016	AAGAA
H017	AAGAC
H018	AAGAG
H019	AAGAT
H020	AAGCA
H021	AAGCC
H022	AAGCG
H023	AAGCT
H024	AAGGA
H025	AAGGC
H026	AAGGT
H027	AAGTA
H028	AAGTC
H029	AAGTG
H030	AAGTT
H031	AATAA
H032	AATAC
H033	AATAG
H034	AATAT
H035	AATCA
H036	AATCC
H037	AATCG
H038	AATCT
H039	AATGA
H040	AATGC
H041	AATGG
H042	AATGT
H043	AATTA
H044	AATTC
H045	AATTG
H046	ACAAC
H047	ACAAG
H048	ACAAT
H049	ACACA
H050	ACACC
H051	ACACG
H052	ACACT

H053	ACAGA
H054	ACAGC
H055	ACAGG
H056	ACAGT
H057	ACATA
H058	ACATC
H059	ACATG
H060	ACATT
H061	ACCAA
H062	ACCAC
H063	ACCAG
H064	ACCAT
H065	ACCCA
H066	ACCCG
H067	ACCCT
H068	ACCGA
H069	ACCGC
H070	ACCGG
H071	ACCGT
H072	ACCTA
H073	ACCTC
H074	ACCTG
H075	ACCTT
H076	ACGAA
H077	ACGAC
H078	ACGAG
H079	ACGAT
H080	ACGCA
H081	ACGCC
H082	ACGCG
H083	ACGCT
H084	ACGGA
H085	ACGGC
H086	ACGGT
H087	ACGTA
H088	ACGTC
H089	ACGTG
H090	ACGTT
H091	ACTAA
H092	ACTAC
H093	ACTAG
H094	ACTAT
H095	ACTCA

H096	ACTCC
H097	ACTCG
H098	ACTCT
H099	ACTGA
H100	ACTGC
H101	ACTGG
H102	ACTGT
H103	ACTTA
H104	ACTTC
H105	ACTTG
H106	AGAAC
H107	AGAAG
H108	AGAAT
H109	AGACA
H110	AGACC
H111	AGACG
H112	AGACT
H113	AGAGA
H114	AGAGC
H115	AGAGG
H116	AGAGT
H117	AGATA
H118	AGATC
H119	AGATG
H120	AGATT
H121	AGCAA
H122	AGCAC
H123	AGCAG
H124	AGCAT
H125	AGCCA
H126	AGCCG
H127	AGCCT
H128	AGCGA
H129	AGCGC
H130	AGCGG
H131	AGCGT
H132	AGCTA
H133	AGCTC
H134	AGCTG
H135	AGCTT
H136	AGGAA
H137	AGGAC
H138	AGGAG

H139	AGGAT
H140	AGGCA
H141	AGGCC
H142	AGGCG
H143	AGGCT
H144	AGGGA
H145	AGGGC
H146	AGGGT
H147	AGGTA
H148	AGGTC
H149	AGGTG
H150	AGGTT
H151	AGTAA
H152	AGTAC
H153	AGTAG
H154	AGTAT
H155	AGTCA
H156	AGTCC
H157	AGTCG
H158	AGTCT
H159	AGTGA
H160	AGTGC
H161	AGTGG
H162	AGTGT
H163	AGTTA
H164	AGTTC
H165	AGTTG
H166	ATAAC
H167	ATAAG
H168	ATAAT
H169	ATACA
H170	ATACC
H171	ATACG
H172	ATACT
H173	ATAGA
H174	ATAGC
H175	ATAGG
H176	ATAGT
H177	ATATA
H178	ATATC
H179	ATATG
H180	ATATT
H181	ATCAA

H182	ATCAC
H183	ATCAG
H184	ATCAT
H185	ATCCA
H186	ATCCG
H187	ATCCT
H188	ATCGA
H189	ATCGC
H190	ATCGG
H191	ATCGT
H192	ATCTA
H193	ATCTC
H194	ATCTG
H195	ATCTT
H196	ATGAA
H197	ATGAC
H198	ATGAG
H199	ATGAT
H200	ATGCA
H201	ATGCC
H202	ATGCG
H203	ATGCT
H204	ATGGA
H205	ATGGC
H206	ATGGT
H207	ATGTA
H208	ATGTC
H209	ATGTG
H210	ATGTT
H211	ATTAA
H212	ATTAC
H213	ATTAG
H214	ATTAT
H215	ATTCA
H216	ATTCC
H217	ATTCG
H218	ATTCT
H219	ATTGA
H220	ATTGC
H221	ATTGG
H222	ATTGT
H223	ATTTA
H224	ATTTC

H225	ATTTG
H226	CAAAC
H227	CAAAG
H228	CAAAT
H229	CAACA
H230	CAACC
H231	CAACG
H232	CAACT
H233	CAAGA
H234	CAAGC
H235	CAAGG
H236	CAAGT
H237	CAATA
H238	CAATC
H239	CAATG
H240	CAATT
H241	CACAA
H242	CACAC
H243	CACAG
H244	CACAT
H245	CACCA
H246	CACCG
H247	CACCT
H248	CACGA
H249	CACGC
H250	CACGG
H251	CACGT
H252	CACTA
H253	CACTC
H254	CACTG
H255	CACTT
H256	CAGAA
H257	CAGAC
H258	CAGAG
H259	CAGAT
H260	CAGCA
H261	CAGCC
H262	CAGCG
H263	CAGCT
H264	CAGGA
H265	CAGGC
H266	CAGGT
H267	CAGTA

H268	CAGTC
H269	CAGTG
H270	CAGTT
H271	CATAA
H272	CATAC
H273	CATAG
H274	CATAT
H275	CATCA
H276	CATCC
H277	CATCG
H278	CATCT
H279	CATGA
H280	CATGC
H281	CATGG
H282	CATGT
H283	CATTA
H284	CATTC
H285	CATTG
H286	CCAAC
H287	CCAAG
H288	CCAAT
H289	CCACA
H290	CCACC
H291	CCACG
H292	CCACT
H293	CCAGA
H294	CCAGC
H295	CCAGG
H296	CCAGT
H297	CCATA
H298	CCATC
H299	CCATG
H300	CCATT
H301	CCGAA
H302	CCGAC
H303	CCGAG
H304	CCGAT
H305	CCGCA
H306	CCGCC
H307	CCGCG
H308	CCGCT
H309	CCGGA
H310	CCGGC

H311	CCGGT
H312	CCGTA
H313	CCGTC
H314	CCGTG
H315	CCGTT
H316	CCTAA
H317	CCTAC
H318	CCTAG
H319	CCTAT
H320	CCTCA
H321	CCTCC
H322	CCTCG
H323	CCTCT
H324	CCTGA
H325	CCTGC
H326	CCTGG
H327	CCTGT
H328	CCTTA
H329	CCTTC
H330	CCTTG
H331	CGAAC
H332	CGAAG
H333	CGAAT
H334	CGACA
H335	CGACC
H336	CGACG
H337	CGACT
H338	CGAGA
H339	CGAGC
H340	CGAGG
H341	CGAGT
H342	CGATA
H343	CGATC
H344	CGATG
H345	CGATT
H346	CGCAA
H347	CGCAC
H348	CGCAG
H349	CGCAT
H350	CGCCA
H351	CGCCG
H352	CGCCT
H353	CGCGA

H354	CGCGC
H355	CGCGG
H356	CGCGT
H357	CGCTA
H358	CGCTC
H359	CGCTG
H360	CGCTT
H361	CGGAA
H362	CGGAC
H363	CGGAG
H364	CGGAT
H365	CGGCA
H366	CGGCC
H367	CGGCG
H368	CGGCT
H369	CGGGA
H370	CGGGC
H371	CGGGT
H372	CGGTA
H373	CGGTC
H374	CGGTG
H375	CGGTT
H376	CGTAA
H377	CGTAC
H378	CGTAG
H379	CGTAT
H380	CGTCA
H381	CGTCC
H382	CGTCG
H383	CGTCT
H384	CGTGA
H385	CGTGC
H386	CGTGG
H387	CGTGT
H388	CGTTA
H389	CGTTC
H390	CGTTG
H391	CTAAC
H392	CTAAG
H393	CTAAT
H394	CTACA
H395	CTACC
H396	CTACG

H397	CTACT
H398	CTAGA
H399	CTAGC
H400	CTAGG
H401	CTAGT
H402	CTATA
H403	CTATC
H404	CTATG
H405	CTATT
H406	CTCAA
H407	CTCAC
H408	CTCAG
H409	CTCAT
H410	CTCCA
H411	CTCCG
H412	CTCCT
H413	CTCGA
H414	CTCGC
H415	CTCGG
H416	CTCGT
H417	CTCTA
H418	CTCTC
H419	CTCTG
H420	CTCTT
H421	CTGAA
H422	CTGAC
H423	CTGAG
H424	CTGAT
H425	CTGCA
H426	CTGCC
H427	CTGCG
H428	CTGCT
H429	CTGGA
H430	CTGGC
H431	CTGGT
H432	CTGTA
H433	CTGTC
H434	CTGTG
H435	CTGTT
H436	CTTAA
H437	CTTAC
H438	CTTAG
H439	CTTAT

H440	CTTCA
H441	CTTCC
H442	CTTCG
H443	CTTCT
H444	CTTGA
H445	CTTGC
H446	CTTGG
H447	CTTGT
H448	CTTTA
H449	CTTTC
H450	CTTTG
H451	GAAAC
H452	GAAAG
H453	GAAAT
H454	GAACA
H455	GAACC
H456	GAACG
H457	GAACT
H458	GAAGA
H459	GAAGC
H460	GAAGG
H461	GAAGT
H462	GAATA
H463	GAATC
H464	GAATG
H465	GAATT
H466	GACAA
H467	GACAC
H468	GACAG
H469	GACAT
H470	GACCA
H471	GACCG
H472	GACCT
H473	GACGA
H474	GACGC
H475	GACGG
H476	GACGT
H477	GACTA
H478	GACTC
H479	GACTG
H480	GACTT
H481	GAGAA
H482	GAGAC

H483	GAGAG
H484	GAGAT
H485	GAGCA
H486	GAGCC
H487	GAGCG
H488	GAGCT
H489	GAGGA
H490	GAGGC
H491	GAGGT
H492	GAGTA
H493	GAGTC
H494	GAGTG
H495	GAGTT
H496	GATAA
H497	GATAC
H498	GATAG
H499	GATAT
H500	GATCA
H501	GATCC
H502	GATCG
H503	GATCT
H504	GATGA
H505	GATGC
H506	GATGG
H507	GATGT
H508	GATTA
H509	GATTC
H510	GATTG
H511	GCAAC
H512	GCAAG
H513	GCAAT
H514	GCACA
H515	GCACC
H516	GCACG
H517	GCACT
H518	GCAGA
H519	GCAGC
H520	GCAGG
H521	GCAGT
H522	GCATA
H523	GCATC
H524	GCATG
H525	GCATT

H526	GCCAA
H527	GCCAC
H528	GCCAG
H529	GCCAT
H530	GCCCA
H531	GCCCG
H532	GCCCT
H533	GCCGA
H534	GCCGC
H535	GCCGG
H536	GCCGT
H537	GCCTA
H538	GCCTC
H539	GCCTG
H540	GCCTT
H541	GCGAA
H542	GCGAC
H543	GCGAG
H544	GCGAT
H545	GCGCA
H546	GCGCC
H547	GCGCG
H548	GCGCT
H549	GCGGA
H550	GCGGC
H551	GCGGT
H552	GCGTA
H553	GCGTC
H554	GCGTG
H555	GCGTT
H556	GCTAA
H557	GCTAC
H558	GCTAG
H559	GCTAT
H560	GCTCA
H561	GCTCC
H562	GCTCG
H563	GCTCT
H564	GCTGA
H565	GCTGC
H566	GCTGG
H567	GCTGT
H568	GCTTA

H569	GCTTC
H570	GCTTG
H571	GGAAC
H572	GGAAG
H573	GGAAT
H574	GGACA
H575	GGACC
H576	GGACG
H577	GGACT
H578	GGAGA
H579	GGAGC
H580	GGAGG
H581	GGAGT
H582	GGATA
H583	GGATC
H584	GGATG
H585	GGATT
H586	GGCAA
H587	GGCAC
H588	GGCAG
H589	GGCAT
H590	GGCCA
H591	GGCCG
H592	GGCCT
H593	GGCGA
H594	GGCGC
H595	GGCGG
H596	GGCGT
H597	GGCTA
H598	GGCTC
H599	GGCTG
H600	GGCTT
H601	GGGAA
H602	GGGAC
H603	GGGAG
H604	GGGAT
H605	GGGCA
H606	GGTAA
H607	GGTAC
H608	GGTAG
H609	GGTAT
H610	GGTCA
H611	GGTCC

H612	GGTCG
H613	GGTCT
H614	GGTGA
H615	GGTGC
H616	GGTGG
H617	GGTGT
H618	GGTTA
H619	GGTTC
H620	GGTTG
H621	GTAAC
H622	GTAAG
H623	GTAAT
H624	GTACA
H625	GTACC
H626	GTACG
H627	GTACT
H628	GTAGA
H629	GTAGC
H630	GTAGG
H631	GTAGT
H632	GTATA
H633	GTATC
H634	GTATG
H635	GTATT
H636	GTCAA
H637	GTCAC
H638	GTCAG
H639	GTCAT
H640	GTCCA
H641	GTCCG
H642	GTCCT
H643	GTCGA
H644	GTCGC
H645	GTCGG
H646	GTCGT
H647	GTCTA
H648	GTCTC
H649	GTCTG
H650	GTCTT
H651	GTGAA
H652	GTGAC
H653	GTGAG
H654	GTGAT

H655	GTGCA
H656	GTGCC
H657	GTGCG
H658	GTGCT
H659	GTGGA
H660	GTGGC
H661	GTGGT
H662	GTGTA
H663	GTGTC
H664	GTGTG
H665	GTGTT
H666	GTTAA
H667	GTTAC
H668	GTTAG
H669	GTTAT
H670	GTTCA
H671	GTTCC
H672	GTTCG
H673	GTTCT
H674	GTTGA
H675	GTTGC
H676	GTTGG
H677	GTTGT
H678	GTTTA
H679	GTTTC
H680	GTTTG
H681	TAAAC
H682	TAAAG
H683	TAAAT
H684	TAACA
H685	TAACC
H686	TAACG
H687	TAACT
H688	TAAGA
H689	TAAGC
H690	TAAGG
H691	TAAGT
H692	TAATA
H693	TAATC
H694	TAATG
H695	TAATT
H696	TACAA
H697	TACAC

H698	TACAG
H699	TACAT
H700	TACCA
H701	TACCG
H702	TACCT
H703	TACGA
H704	TACGC
H705	TACGG
H706	TACGT
H707	TACTA
H708	TACTC
H709	TACTG
H710	TACTT
H711	TAGAA
H712	TAGAC
H713	TAGAG
H714	TAGAT
H715	TAGCA
H716	TAGCC
H717	TAGCG
H718	TAGCT
H719	TAGGA
H720	TAGGC
H721	TAGGT
H722	TAGTA
H723	TAGTC
H724	TAGTG
H725	TAGTT
H726	TATAA
H727	TATAC
H728	TATAG
H729	TATAT
H730	TATCA
H731	TATCC
H732	TATCG
H733	TATCT
H734	TATGA
H735	TATGC
H736	TATGG
H737	TATGT
H738	TATTA
H739	TATTC
H740	TATTG

H741	TCAAC
H742	TCAAG
H743	TCAAT
H744	TCACA
H745	TCACC
H746	TCACG
H747	TCACT
H748	TCAGA
H749	TCAGC
H750	TCAGG
H751	TCAGT
H752	TCATA
H753	TCATC
H754	TCATG
H755	TCATT
H756	TCCAA
H757	TCCAC
H758	TCCAG
H759	TCCAT
H760	TCCCA
H761	TCCCG
H762	TCCCT
H763	TCCGA
H764	TCCGC
H765	TCCGG
H766	TCCGT
H767	TCCTA
H768	TCCTC
H769	TCCTG
H770	TCCTT
H771	TCGAA
H772	TCGAC
H773	TCGAG
H774	TCGAT
H775	TCGCA
H776	TCGCC
H777	TCGCG
H778	TCGCT
H779	TCGGA
H780	TCGGC
H781	TCGGT
H782	TCGTA
H783	TCGTC

H784	TCGTG
H785	TCGTT
H786	TCTAA
H787	TCTAC
H788	TCTAG
H789	TCTAT
H790	TCTCA
H791	TCTCC
H792	TCTCG
H793	TCTCT
H794	TCTGA
H795	TCTGC
H796	TCTGG
H797	TCTGT
H798	TCTTA
H799	TCTTC
H800	TCTTG
H801	TGAAC
H802	TGAAG
H803	TGAAT
H804	TGACA
H805	TGACC
H806	TGACG
H807	TGACT
H808	TGAGA
H809	TGAGC
H810	TGAGG
H811	TGAGT
H812	TGATA
H813	TGATC
H814	TGATG
H815	TGATT
H816	TGCAA
H817	TGCAC
H818	TGCAG
H819	TGCAT
H820	TGCCA
H821	TGCCG
H822	TGCCT
H823	TGCGA
H824	TGCGC
H825	TGCGG
H826	TGCGT

H827	TGCTA
H828	TGCTC
H829	TGCTG
H830	TGCTT
H831	TGGAA
H832	TGGAC
H833	TGGAG
H834	TGGAT
H835	TGGCA
H836	TGGCC
H837	TGGCG
H838	TGGCT
H839	TGGGA
H840	TGGGC
H841	TGGGT
H842	TGGTA
H843	TGGTC
H844	TGGTG
H845	TGGTT
H846	TGTAA
H847	TGTAC
H848	TGTAG
H849	TGTAT
H850	TGTCA
H851	TGTCC
H852	TGTCG
H853	TGTCT
H854	TGTGA
H855	TGTGC
H856	TGTGG
H857	TGTGT
H858	TGTTA
H859	TGTTC
H860	TGTTG
H861	TTAAC
H862	TTAAG
H863	TTAAT
H864	TTACA
H865	TTACC
H866	TTACG
H867	TTACT
H868	TTAGA
H869	TTAGC

H870	TTAGG
H871	TTAGT
H872	TTATA
H873	TTATC
H874	TTATG
H875	TTATT
H876	TTCAA
H877	TTCAC
H878	TTCAG
H879	TTCAT
H880	TTCCA
H881	TTCCG
H882	TTCCT
H883	TTCGA
H884	TTCGC
H885	TTCGG
H886	TTCGT
H887	TTCTA
H888	TTCTC
H889	TTCTG
H890	TTCTT
H891	TTGAA
H892	TTGAC
H893	TTGAG
H894	TTGAT
H895	TTGCA
H896	TTGCC
H897	TTGCG
H898	TTGCT
H899	TTGGA
H900	TTGGC
H901	TTGGT
H902	TTGTA
H903	TTGTC
H904	TTGTG
H905	TTGTT
H906	TTTAA
H907	TGTCT
H908	TGTGA
H909	TGTGC
H910	TGTGG
H911	TGTGT
H912	TGTTA

H913	TGTTC
H914	TGTTG
H915	TGTTT
H916	TTAAC
H917	TTAAG
H918	TTAAT
H919	TTACA
H920	TTACC
H921	TTACG
H922	TTACT
H923	TTAGA
H924	TTAGC
H925	TTAGG
H926	TTAGT
H927	TTATA
H928	TTATC
H929	TTATG
H930	TTATT
H931	TTCAC
H932	TTCAG
H933	TTCAT
H934	TTCCA
H935	TTCCC
H936	TTCCG
H937	TTCCT
H938	TTCGA
H939	TTCGC
H940	TTCGG
H941	TTCGT
H942	TTCTA
H943	TTCTC
H944	TTCTG
H945	TTCTT
H946	TTGAC
H947	TTGAG
H948	TTGAT
H949	TTGCA
H950	TTGCC
H951	TTGCG
H952	TTGCT
H953	TTGGA
H954	TTGGC
H955	TTGGG

H956	TTGGT
H957	TTGTA
H958	TTGTC
H959	TTGTG
H960	TTGTT
